# Bayesian Selection of Markov Models for Symbol Sequences: Application to Microsaccadic Eye Movements

**DOI:** 10.1371/journal.pone.0043388

**Published:** 2012-09-06

**Authors:** Mario Bettenbühl, Marco Rusconi, Ralf Engbert, Matthias Holschneider

**Affiliations:** 1 Institute of Mathematics, Focus Area for Dynamics of Complex Systems, University of Potsdam, Potsdam, Germany; 2 Department of Psychology, DFG Research Unit 868 “Computational Modeling of Behavioral, Cognitive, and Neural Dynamics”, University of Potsdam, Potsdam, Germany; University of Manchester, United Kingdom

## Abstract

Complex biological dynamics often generate sequences of discrete events which can be described as a Markov process. The order of the underlying Markovian stochastic process is fundamental for characterizing statistical dependencies within sequences. As an example for this class of biological systems, we investigate the Markov order of sequences of microsaccadic eye movements from human observers. We calculate the integrated likelihood of a given sequence for various orders of the Markov process and use this in a Bayesian framework for statistical inference on the Markov order. Our analysis shows that data from most participants are best explained by a first-order Markov process. This is compatible with recent findings of a statistical coupling of subsequent microsaccade orientations. Our method might prove to be useful for a broad class of biological systems.

## Introduction

Many biological systems produce discrete sequences of events that can be used to characterize the underlying generating processes, e.g., neural spike trains [1] or saccadic eye movements [2]. Using a coarse-grained description of the data as symbol sequences [3], we can analyze their statistical properties in terms of a Markov process [4]. A critical parameter in such a model is the order of the Markov process which captures the time span of the statistical dependence within the sequence of symbols. Here we propose a Bayesian approach to estimate the order of the underlying Markov process from experimental data.

**Figure 1:**
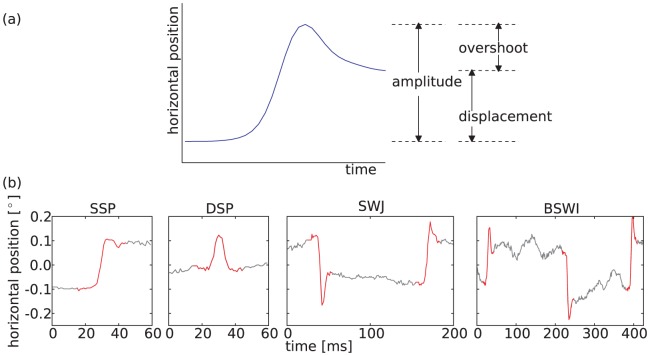
Illustration of microsaccade shape properties reported microsaccade sequence patterns. (a) illustrates a typical microsaccade shape occurring during fixational eye movements with the designated microsaccade properties. (b) shows microsaccade sequence patterns, composed of one, two or three subsequent microsaccadic events, so-called *saccadic intrusions* (SI). From left: single saccadic pulse (SSP), double saccadic pulse (DSP), square-wave jerks (SWJ), biphasic square wave intrusion (BSWI). All patterns have been hand-picked from the horizontal trajectories of fixational eye movements. The separating time intervals are not representative for all participants.

Visual perception with high acuity is based on accurate fixation of a target object. However, our eyes are never motionless and continually produce small irregular movements. Two components of these miniature or fixational eye movements (FEM) are microsaccades (rapid small-amplitude movements) and physiological drift (a slower, random component of the motion) [5]–[8]. Following earlier attempts [9], recent progress has identified basic principles for theoretical models of the generation of fixational eye movements [10]–[15]. First, physiological drift might be described by fractional Brownian motion with two scaling regimes corresponding to persistent and anti-persistent behavior on smaller and larger time scales respectively [12], [16]. Second, microsaccades represent a more ballistic movement type [10], [17]. For an illustration of characteristic microsaccade properties, see Figure 1a.

Hypotheses on the generating mechanism of microsaccades are potentially relevant to the analysis of correlations within sequences of microsaccades. Since sequences of microsaccades appear to have some non-random structure, isolated microsaccades are often distinguished from saccadic intrusions (SI) or square-wave jerks (SWJ) and biphasic square-wave intrusions (BSWI; cf. Fig. 1b). For example, Abadi and Gowen [18], [19] exploited the direction dissimilarity of microsaccadic events and their temporal proximity to define different types of SIs with characteristic kinematic properties (amplitude, displacement) and rate-of-occurrence. As a result of such a classification scheme, isolated microsaccades and SWJ represent the most common type of SIs in healthy humans [18].

The properties of SWJ are highly relevant to neurological disorders [20]–[23]. Recently, Otero-Millan et al. [24] introduced an advanced treatment of microsaccade sequences. Based on a velocity-threshold algorithm [25], Otero-Millan et al. [24] used direction dissimilarity, magnitude dissimilarity, and temporal proximity to calculate a square-wave jerk index which allows a separation of single-standing microsaccades and SWJ. In their study with Progressive Supranuclear Palsy (PSP) patients, Otero-Millan et al. ([24], p. 4386) concluded “that microsaccades and SIs are essentially the same phenomena and that SWJ are generated by a common coupling mechanism in PSP patients and healthy observers.”

Here, we will follow up Otero-Millan et al.'s [24] work using an explicit statistical model of the SWJ coupling mechanism. In our approach, FEM data is coarse-grained by a mapping to discrete sequences of symbols, where each symbol represents a microsaccade orientation. For a first approximation, the elapsed time between microsaccadic events is not taken into account for the statistical description of symbol sequences. Markov chain models of three different orders will be considered: Markov chains of zeroth-, first-, and second-order. They correspond to uncorrelated, one-, and two-lag memory stochastic processes. For the Markov chain of zeroth-order, sequences of microsaccadic shapes are uncorrelated and both SWJ and biphasic square-wave intrusion (cf. Fig. 1b) would occur by chance as sequences of successive single saccadic pulses (cf. Fig. 1b). In contrast, in a Markov chain of first order, pairs of saccadic shapes would be statistically dependent and the chain would also account for SWJ. Finally, in a second-order process, also triplets of saccadic shape would be correlated and a model with memory would also account for BSWI.

In our analysis, we use a Bayesian approach for the estimation of the order of the underlying Markov process from experimentally observed FEM data. Our method will be tested on simulated data with known Markov order and, finally, it will be applied to FEM data of human observers. We will discuss our results with respect to existing frameworks for the analysis of microsaccades.

## Methods

In this section, we give a brief overview on definitions and properties of Markov processes. We then present our symbolic dynamics approach for sequences of microsaccade shapes and the Bayesian estimation of the Markov order. Finally, we shortly summarize the methods used to detect microsaccades and to characterize their shapes [26].

### Stochastic modeling with Markov processes

Consider a sequence of symbols taken from some finite state space 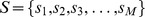. We denote the sequence of states by 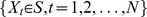. A probability measure *Pr* on the space of such sequences:
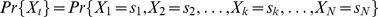
(1)is an *n*-th order Markov measure if it satisfies for all 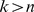:
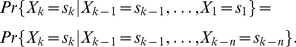
(2)This means that occurrence of a symbol at position *k* depends on the *n* previous symbols only. The memory has the finite length *n* and the probability of any future behavior is not influenced by additional knowledge concerning its past behavior beyond the memory horizon *n* [27], [28]. A stationary Markov chain is a Markov chain, where the transition probability does not explicitly depend on *k* but only on the previous symbols 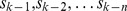 and the new symbol 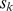. In this work, we consider stationary Markov chains only.

The conditional probabilities in Equation (2) do not fully specify the Markov probability measure in the space of symbol sequences. We have to specify in addition the initial distribution:
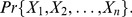
(3)We say the measure is (shift)invariant if the distribution of symbols remains the same under the Markov dynamics given by Equation (2):
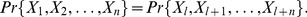
(4)In the generic case, there is only one invariant measure for a given dynamic. This is the probability model of sequences that we consider. In particular, we are now interested in the estimation of the order of the underlying process.

If we introduce the space 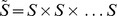 (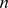-factors) of compound symbols of order *n*, or words of length *n* in the alphabet *S*, then the *n*-th order Markov chain process is representable as a first-order Markov process in the symbol space 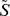. However, many transitions in this extended space are not possible and will enter the transition matrix as *0*s.

Here, we investigate processes up to an order of three for a symbol space 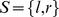. In case of a zeroth-order Markov chain, the past has no influence and each new symbol is independently drawn from a probability distribution *Pr* over the symbol space *S*. Therefore such a process is described by a single number 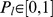, which is the probability to draw *l*. The unique invariant measure is induced through 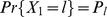.

In the case of a first-order Markov chain in two symbols we write for a transition from *l* to *r* in a time step 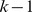 to *k*:
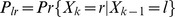
(5)and obtain the transition matrix 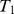 as:
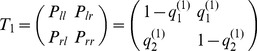
(6)Note that everything is parameterized in terms of the two numbers 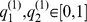. The shift-invariant distribution 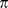 for a first-order Markov process [28] is now determined through the left-hand or Perron-Frobenius eigenvector of the transition matrix [29]. This eigenvector 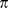 solves the equation:
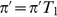
(7)with 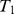 the transition matrix, 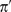 the transpose of the column eigenvector, representing the probabilities of the stationary distribution, with 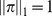 and all entries 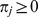.

For a second order chain, we have to consider all the transitions from the last two symbols to the new symbol. To fix the ideas consider a transition from *lr* to *r*:
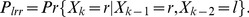
(8)This defines 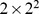 numbers of which only 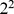 are independent, since e.g. 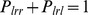. This process is equivalent to a first order process in the space of words of length 2 by identifying the transition from *ab* to *c* (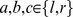) with the compound transition *ab* to *bc*. However, a transition *ll* to *rr* is impossible and therefore the associated transition matrix 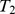 is 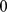 for these transitions. In terms of the numbers 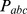 of Equation (8) the transition matrix 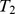 reads:
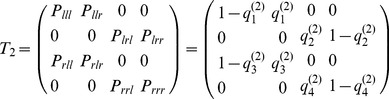
(9)Again, everything is parameterized by the four numbers 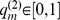 for 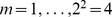. The stationary distribution can be computed as the Perron-Frobenius eigenvector analogous equation (Eq. 7).

### Bayesian model selection

For an observed data set *D*, a collection of parameterized stochastic models can be proposed and the question is which model fits the best. The selection criteria should not only take into account how well the data is described but also the complexity of the model. The goal is not only to optimally fit existing data, but also to minimize the future prediction error when new unobserved data becomes available. Optimizing only the fit leads to poor prediction performance, a phenomenon known as overfitting the data. In the Bayesian setting, the model selection is commonly done with the help of the Bayes factor [30]. For a data set *D*, the Bayes factor for comparison between the alternative models 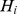 and 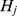 is defined by:
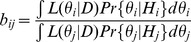
(10)which is the ratio of the integrated posterior, i.e., regarding Bayes theorem for probability densities, it is the ratio of evidences [30]. Here, 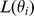 refers to the likelihood of the parameter set 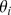 which parameterizes a model under hypothesis 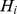, given a data set *D*. The probability 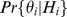 is the prior for the parameter set 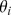 under hypothesis 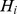. The same notation is used for 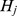.

To discriminate even smaller differences between hypotheses, we log-transform Equation (10) to deciban scale:
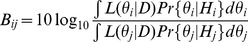
(11)The value of 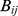 provides evidence in favor of one hypothesis against the other. Values of 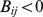 will support the null hypothesis 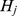, otherwise, values in the range 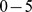, 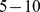, 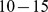, 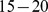, and larger than 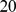 provide weak, substantial, strong, very strong, and decisive evidence against 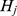, respectively (cf. [31]).

### The Markov model for microsaccades

The question arises how we could estimate the order of the Markov chain by combining knowledge about Markov processes, Bayes factor, and symbolic dynamics. If we assume that sequences of microsaccade directions — with *l* and *r* representing leftward and rightward microsaccades — can be described by Markov chains, then it is the Markov order which we need to estimate. The parameter space of an 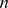
th-order Markov chain model is 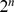 dimensional. We denote these parameters by 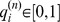, 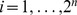. As a shortcut for all parameters of an 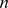
th-order Markov chain model we use 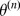. We propose to use the Bayes factor analysis to discriminate between different orders of chains. Under the assumption that a given sequence 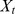, 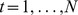 of microsaccade directions, 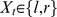, can be described by a discrete-time stationary 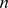
th-order Markov process, we can write the likelihood of a sequence 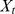 given the transition probabilities as:
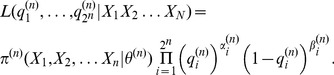
(12)Here, 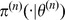 is the probability to find the first *n* symbols in the stationary distribution. This stationary distribution itself depends on the parameters 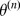. On the remaining symbols 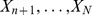, the numbers 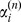, 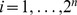, count for each word (enumerated by *i*) the number of transitions for which the new symbol that is added to the sequence was different from the last symbol in the word, whereas 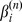 counts the transitions from word 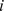 to the last symbol of 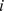. Since the likelihood function for the whole data 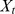 depends only on these numbers and the first *n* symbols, this data represents a sufficient statistic. Therefore, all information about the underlying process is contained in these numbers.

For independent realizations, we can take the product of the individual likelihood functions. In this case, the individual counts for each trial simply add up to the total counts. The probabilities of the initial sequences, however, have to be multiplied.

As prior information about the transition probabilities 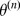 we chose a flat (constant) prior in a hypercube of dimension 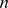. Based on the calculation of the likelihood of a particular *n*th-order Markov chain, we can compare Markov chains of different orders using the Bayes factor (Eq. 11).

Highly important for the estimation of the Bayes factor are the integrations in Equation (11). In our work we assumed that the sequences of microsaccades directions are realizations of a stationary stochastic process that we described as a Markov process with unknown order. If one has long sequences, containing a large number of symbols, the prefactor 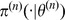 could be assumed to be uniformly distributed. This assumption is equivalent to assigning the probability 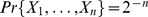 to any initial state of the *n*th-order Markov chain. In this case, the integrals in Equation (11) are analytically solvable by a product of Beta functions 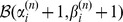 with 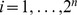 and *n* the order of the Markov chain. For sequences of symbols representing microsaccade directions in a 20 second time interval, the number of symbols is between 20 and 50 [26], leaving us with short sequences. In this case, the term 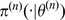 remains in the integral and has to be computed numerically for each different order of the Markov chain. To obtain the integrated likelihood through numerical integration, the midpoint rule was used with 100 equidistantly sampled integration points between 0 and 1, for each integral of 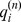. For each value of the parameters 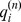, we evaluated the transition matrix of the process and computed the Perron-Frobenius eigenvector, the stationary distribution of the process. The probability of the initial state of the chain is then drawn from this stationary distribution 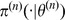.

### Simulation of Markov chains

To verify our method, we simulated two-states Markov chains of zeroth-, first-, and second-order. We generated 532 sequences for each different Markov order, using 19 different random transition matrices and comparable sequence lengths as for the microsaccade sequences. We first computed 30,000 iterates to remove transients of the process and then chose sequences of different lengths.

### Video-based eye tracking in a fixation task

Data used in our study were previously published in [12], [32]. Human participants with an average age of 22 years and normal or corrected-to-normal vision performed a fixation task. A black square on white background (3×3 pixels on a computer display which corresponds to a spatial extent of 7.2 arcmin; Iiyama, Vision Master Pro 514, 40 by 30 cm, 100 Hz, 1024×768 pixels) was presented to the participants. Participants were asked to fixate this point. Each subject was required to perform 30 trials (20 seconds each) and was asked to prevent blinking during each trial. An online check for blinks was applied. To avoid false detection of blinks, we checked the trajectories by hand and skipped trials in which a blink occurred. The number of trials that entered the analysis for each participant is reported in Table 1. Every fixation trial was followed by a presentation of a photograph for 10 seconds, allowing participants to relax and perform inspection saccades or blinks. At the beginning of each trial, participants performed a 9-point calibration. The luminance of the screen was kept constant during both, calibration and recording. This prevented changes of pupil size due to luminance variation. The trajectories were recorded using a head mounted eye-tracking system (EyeLink II, SR Research, Osgoode, Ontario, Canada), which generated binocular recordings of eye movements at a sampling rate of 500 Hz. The spatial resolution for a dark pupil was higher than 0.01° (RMS) visual angle. Participants were seated on a chair at 50 cm viewing distance. To reduce body and head movements, a chin rest was used. The experiment was performed in accordance with the declaration of Helsinki.

**Table 1:** Microsaccade properties and estimated Markov order.

Subject	No. of trials	Rate [MS/ 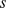 ]	Ampl. [°]	Displ. [°]	Estimated Markov order	No. of microsaccades	IMSI [ 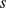 ]
01	30	0.74	0.21	0.1	first	252 - 191	1.35
02	30	1.12	0.34	0.24	second	409 - 265	0.88
03	22	0.42	0.2	0.15	zeroth	47 - 139	2.29
04	30	1.26	0.23	0.15	first	210 - 547	0.78
05	30	1.34	0.24	0.16	first	355 - 448	0.75
06	30	1.48	0.37	0.29	second	382 - 503	0.7
07	17	0.73	0.16	0.12	first	103 - 146	1.35
08	28	0.84	0.4	0.34	first	261 - 210	1.24
09	30	0.6	0.51	0.34	first	147 - 214	1.46
10	30	0.72	0.13	0.08	first	270 - 160	1.33
11	29	1.37	0.22	0.16	first	483 - 311	0.79
12	30	0.78	0.16	0.11	first	336 - 129	1.34
13	29	0.37	0.17	0.11	first	141 - 73	2.67
14	23	0.99	0.39	0.21	zeroth	194 - 262	1.01
15	29	1.31	0.27	0.16	first	315 - 445	0.79
16	29	1.7	0.27	0.22	first	435 - 550	0.58
17	29	0.84	0.32	0.22	zeroth	96 - 393	1.23
18	30	1.2	0.14	0.09	second	526 - 193	0.82
19	29	0.87	0.56	0.44	first	236 - 271	1.01
total	534	0.98	0.21	0.14	first	5198 - 5450	0.99

Microsaccade and microsaccade sequence properties for the left eye's movements of nineteen participants in a fixation task experiment. Amplitude (Ampl.), displacement (Displ.), rate and IMSI are given as average over all trials of the participant that entered the analysis (No. of trials). Number of microsaccades are given as left- and rightward directed.

### Microsaccade detection and characterization

Mergenthaler and Engbert [12] have shown that FEM can statistically be described by a self-similar process, in this case modeled by fractional Brownian motion. Our detection method uses the influence of microsaccades on the self-similarity of the FEM's drift to detect the former in the time series. This method, introduced in [26], is based on the continuous wavelet transform to detect events of less self-similar behavior. Microsaccades are then defined as binocularly occurring singularities. In the same work, we had introduced a two-component model of the microsaccade shape, which resulted from the principal component analysis. We characterize microsaccades as binocular events whose variance in shape can be described by at least 80% with the microsaccade shape model [26].

**Figure 2:**
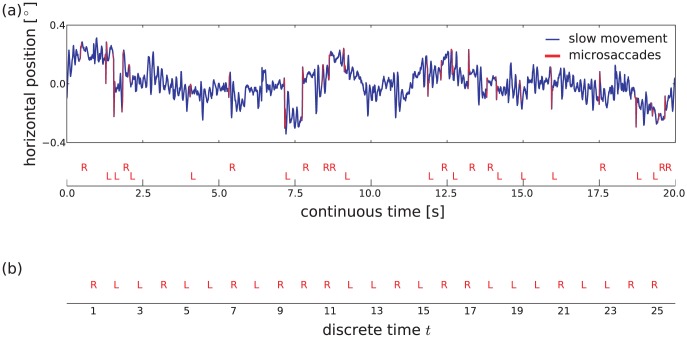
Horizontal FEM trajectory with detected microsaccades and illustration of the sequence of microsaccade directions. (a) Trajectory of a 20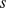 FEM trial with (*upper panel*) detected microsaccades and (*lower panel*) directions of microsaccades. (b) Sequence of microsaccade directions represented as discrete time series of binary states. For the analysis of microsaccade direction sequences, we neglect the temporal proximity existent in the sequence of microsaccades.

To detect the direction of a microsaccadic eye movement, we mapped the snippet of the trajectory that corresponds to a microsaccade onto the two-component model. Depending on the sign of the coefficient for the first component, we determine the direction as *R*ight or *L*eft, for positive or negative sign, respectively. Considering the direction of microsaccades and neglecting temporal proximity, we mapped the sequence of microsaccades into a time-discrete sequence of two symbols *L*eft and *R*ight, as shown in Figure 2.

## Results

### Exact Bayesian estimation of Markov order

Using the method introduced above, we generated sequences from Markov chains of zeroth-, first-, and second-order. By definition, the zeroth-order Markov chain is an uncorrelated random process. After the computation of 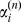 and 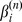, we used Equation (11) and (12) to estimate the order of the chain.

**Figure 3:**
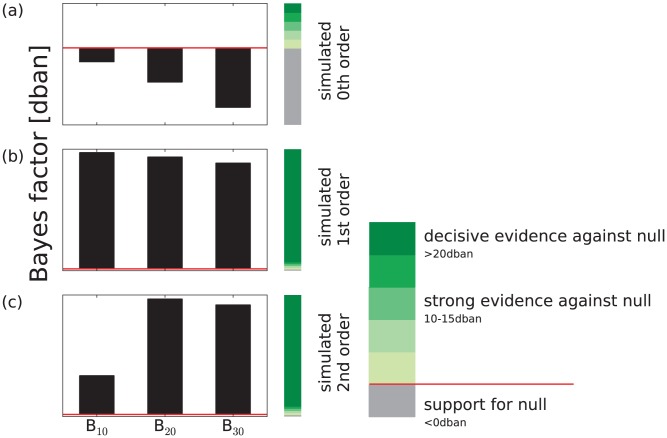
Markov order estimation for sequences of simulated different order Markov chains. Using a parameterization as zeroth-order Markov chain as null hypothesis, we compared in the Bayes factor the evidences against first-, second-, and third-order parameterization of Markov chain. We simulated sequences of: (a) uncorrelated random processes, (b) first-order Markov chain, and (c) second-order Markov chains, each of two symbols. In (a) we obtained support for zeroth-order parameterization, in (b) evidence against the null for all orders but highest with a first-order parameterization and (c) accordingly for a second-order parameterization. This validated the estimator to be correct.

This was done by evaluating the Bayes factor (Eq. 11) and using a flat prior. Due to the hierarchical structure of the Markov models, this approach is an application of Occam's razor in ambiguous conditions: when the Bayes factor supports two alternative hypotheses with equivalent strength, the most parsimonious model, i.e., the lowest order, would be selected. We present in Figure 3 the results of the Bayes factor analysis, which we obtained by means of Monte Carlo simulation, for simulations of sequences of Markov chains of (a) zeroth-, (b) first-, and (c) second-order.

Throughout our Bayes factor analysis, the parameterization which led to a zeroth-order Markov chain is taken as null hypothesis. For simulated uncorrelated random processes, the estimation returned a zeroth-order Markov chain as the best descriptor for the data. None of the hypotheses of higher-order Markov chains showed evidence against the null hypothesis (cf. Fig. 3a). For sequences simulated from a first-order Markov chain, the estimator presented the highest evidence against the null hypothesis for the first-order parameterization. Due to nesting of the Markov chains, a parameterization as second- or third-order Markov chain also puts evidence against the null hypothesis, i.e. the zeroth-order parameterization. But the scale of interpretation of the Bayes factor allows the separation of the first-order Markov chain from the others. The Bayes factor analysis estimated the parameterization of the Markov chain as first-order (cf. Fig. 3b). We obtained a similar result for the simulated sequences of second-order Markov chains. Here, the highest evidence against the null is given for a second-order parameterization of the Markov chain model (cf. Fig. 3c). The order was estimated correctly.

### The dynamical Markov model of microsaccade directions

Using the direction of the horizontal component of the microsaccadic eye movements, we obtained sequences of 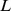
eft and 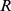
ight movements, as illustrated in Figure 2. Under the assumption that microsaccade direction sequences of an individual subject can be evaluated as realizations of a single Markov process, we summed the counts 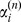 and 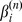 from all different trials of one participant and estimated the order of the Markov chain as described above.

**Figure 4:**
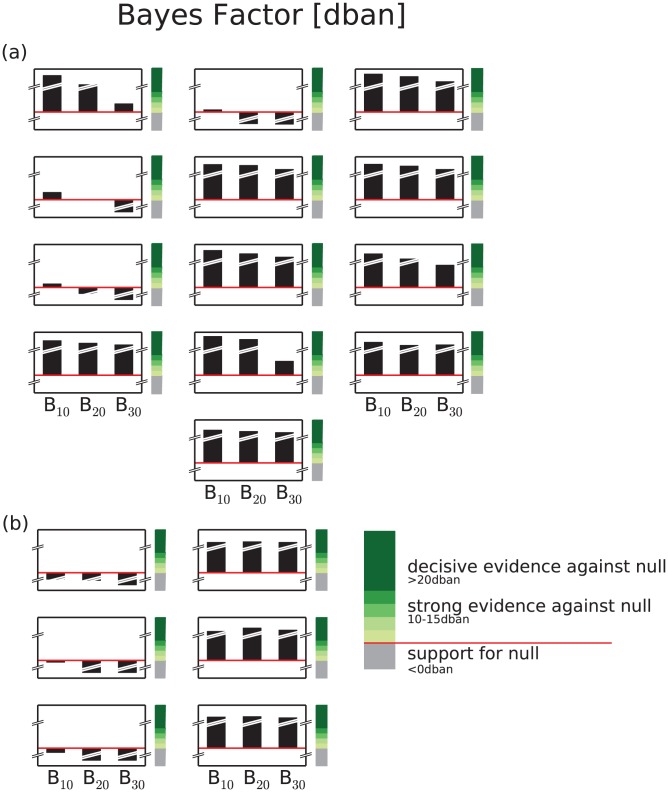
Markov order estimation for microsaccade sequences of nineteen participants in a fixation task experiment. Using the zeroth-order parameterization of the Markov chain model as null hypothesis, we calculated the Bayes factor to separate that order which best described the sequences of microsaccade directions. (a) The thirteen participants show evidence of different strengths against the null hypothesis. Through parsimony, a first-order parameterization of the Markov chain would be estimated as best descriptor. (b) For six participants, support for zeroth- (left column) or second-order (right column) is estimated.

Selecting the null hypothesis of a zeroth-order Markov chain, we compare the parameterizations, corresponding to higher-order Markov chains, against this null hypothesis. For thirteen participants, the estimator preferred the parameterization as first-order Markov chain, i.e., compared against zeroth-order parameterization, evidence against the latter is found (cf. Fig. 4a). Although in comparison with second- and third-order parameterization, the null hypothesis was supported, it is the nesting of Markov chains and additionally the scale of interpretation of the Bayes factor that revealed the first-order parameterization of the Markov chain model as the best estimate. In Figure 4b, the analysis is presented for the remaining 6 out of 19 participants. For three subjects, no evidence against the null hypothesis, i.e., against a parameterization as zeroth-order Markov chain existed (cf. Fig. 4b, left column). The microsaccade sequences mapped on the sequences of two symbols could be described best for the remaining three subjects by a second-order Markov chain. The highest evidence against the null hypothesis was calculated for a second-order parameterization (cf. Fig. 4b, right column).

Yet, for thirteen participants, a parameterization of the model as first-order Markov chain returns the best fitting stochastic process for the microsaccade sequences. Nevertheless, for six participants, the support for zeroth- or second-order parameterization of the Markov chain is very close to be supportive for first-order Markov chain parameterization as well.

### Relationship between microsaccade properties and estimated Markov order

Table 1 summarizes for each participant the estimated order of the Markov chain and the characteristic properties of both, microsaccades and microsaccades sequences. The part of the time series that corresponds to a microsaccadic event is mapped on the two-components model [26]. The amplitude and displacement were evaluated for the horizontal component of the microsaccade. The average rate and intermicrosaccade intervals (IMSI) were taken from those microsaccadic events that are binocularly appearing in both eyes and whose variance can be mapped on the two components of the microsaccade model by at least 80%.

**Figure 5:**
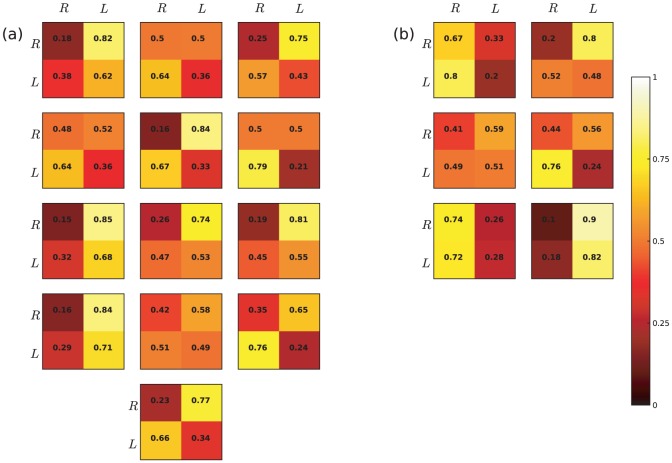
Each box represents the transition matrix for each participants. Participants are ordered as in Figure 4. The values are color-coded to facilitate reading. Only the transition matrix for a first order Markov chain is reported.

Figure 5 shows the one-step transition matrices for each participant for a first-order Markov model. The values are calculated on the maximum likelihood estimates, i.e.:
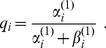
(13)There are inter-individual differences for transition matrices across participants. We identified participants for which the probabilities to change or stay in a state are almost equal. But still a first-order Markov chain describes their sequences best although the probabilities are close to 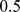, i.e., chance-level in uncorrelated random processes. Furthermore, the same evidences against higher- or lower-order Markov chains does not imply that the transition matrices are similar, too. Therefore, a sole analysis on the transition matrices to determine the Markov order would not give correct results.

## Discussion

Symbol sequences obtained from experimental observations of complex biological dynamics can be modeled by a stochastic process. We developed a new procedure for the estimation of the order of an underlying Markov process. The framework was developed and tested for simulated data and applied to microsaccadic eye movements from human eye tracking recordings.

### Bayesian estimation of the Markov order

Before investigating data from human fixation, we tested our method on simulated data. We generated realizations of zeroth-, first- and second-order Markov chains and we inferred their order. We compared the evidences for parameterization as higher-order Markov chains against the null hypothesis which referred to a parameterization as zeroth-order Markov chain.

Although different order Markov chains are nested into each other, we were able to recover the correct order of each simulated data set. The scale of interpretation of the Bayes factor analysis lets us even separate models with smallest differences — here, a Markov model with different parameterizations, i.e., different orders. The estimator turned out to be useful with a flat prior on model probabilities, since more complex hypotheses, i.e., higher-order Markov chains, contain a larger variance in their marginal likelihoods.

### Symbolic dynamics for sequences of microsaccades

As a first step, we derived symbol sequences from microsaccadic eye movements by a coarse-graining strategy. We neglected temporal intervals between subsequent events and mapped microsaccades to symbols based on their spatial orientation. Despite this simplification, the resulting symbol sequences kept robust properties of the experimental data. In particular, we were interested in the statistical dependence between the orientations in sequences of microsaccades. For each given sequence, we computed the transition probability matrix for each participant. Transition probabilities were used for likelihood computations within the framework of Bayesian inference.

### A Markov model for microsaccade sequences

Earlier findings in the analysis of microsaccades postulate the existence of a statistical coupling of subsequent microsaccade orientations, e.g., square-wave jerks (SWJ) and biphasic square wave intrusions (BSWI) [18], in addition to isolated microsaccades. Under the assumption that sequences of microsaccade directions are realizations of a discrete-time Markov process, we estimate the order of the chain from experimental data. A zeroth-order Markov chain represents a stationary stochastic process without any memory of the current state, i.e., an uncorrelated process. In contrast, the first-order Markov chain could produce SWJ, i.e., sequence of two microsaccades with opposite directions, and isolated microsaccades as result of the same process with one-step memory, i.e., when knowledge of the current state influences the probability for the upcoming transition. The sequences realized in a BSWI can be explained as follows: The first microsaccade drives the eye away from its initial position and the next two microsaccades return the eye to its launch site, i.e., the third microsaccade depends on the previous two microsaccadic events as the second event was not error-correcting but overshot the launch site. However, according to our analyses using the Bayes factor, the first-order Markov chain is the best description of most of the experimentally observed microsaccade sequences. Thus, the observed pattern of a BSWI occurs only by chance in a process with memory length 1 such that statistically, no support is given for the existence of such pattern if not occurring randomly. This adds to the statistical description of the microsaccade dynamics and lends support to “the idea of a continuum between microsaccades and SIs (or at least microsaccades and SWJ)” as proposed by Otero-Millan et al. ([24], p. 4385), following the original idea of Gowen et al. ([33], p. 154).

Moreover, Otero-Millan et al. concluded “that microsaccades and SIs are essentially the same phenomena” ([24], p. 4386) but highlighted before that “Future studies should investigate the relationship between microsaccades and other types of saccadic intrusions” ([24], p. 4385). Following [18], these other types of saccadic eye movements are single saccadic pulse (SSP), double saccadic pulse (DSP), and biphasic square wave intrusion (BSWI). Having obtained strong evidence for a one-step memory process as model for the generation of microsaccades and SWJ, the BSWI component might be interpreted as a rare random event in the sequence of directions with first-order statistical dependence. The second-order Markov chain with its two-step memory is compatible with the data of only three participants, however, the scale of interpretation of the Bayes factor yielded only very weak support against a first-order Markov chain, if a direct comparison is made.

In our analysis, we considered the dynamics of the microsaccade directions, disrespecting a possible coupling between physiological drift and microsaccades [6], [10], [34]. Further analyses, which combine both components of fixational eye movements, could potentially lead to more complicated process assumptions.

A potential application of our method rises from the observation that microsaccade directions seem to be influenced by the relative eye position and fixation target. Otero et al. [24] showed for a subgroup of participants, whose eye movements have been recorded with the scleral search coil technique [35], that the first microsaccade in a SWJ is error-producing. It may be possible that in a region which is close to the fixated target, microsaccades are error-producing and thus independent from the previous direction. On the other hand, microsaccades triggered far away from the target may be error-correcting and thus related to the previous saccadic direction. In this scheme, it may be possible to discriminate between two separate regions around the fixation target: a nearby region where the SWJ are the majority and thus the sequences of microsaccade shapes are better described by a Markov chain of first-order and a far-away region in which it may be possible to find a more complex behavior leading to a mixture of microsaccade shapes. For our analysis, limitations of the EyeLink II system to resolve the absolute eye position with necessary accuracy of more than 0.5° do not allow us to perform a comparable analysis with our data. But it should be considered in studies in which the resolution is higher.

While we demonstrated our method on examples from fixational eye movements, we believe that the Bayesian estimation of the Markov order of a stochastic process underlying the generation of symbol sequences will turn out as a powerful tool for a broad range of biological systems.
